# Plant biodiversity assessment through pollen DNA metabarcoding in Natura 2000 habitats (Italian Alps)

**DOI:** 10.1038/s41598-021-97619-3

**Published:** 2021-09-14

**Authors:** Kleopatra Leontidou, Despoina Vokou, Anna Sandionigi, Antonia Bruno, Maria Lazarina, Johannes De Groeve, Mingai Li, Claudio Varotto, Matteo Girardi, Maurizio Casiraghi, Antonella Cristofori

**Affiliations:** 1 Department of Biodiversity and Molecular Ecology, Research and Innovation Centre, Fondazione Edmund Mach, Via E. Mach 1, 38010 San Michele all’ Adige, Trentino Italy; 2 Department of Ecology, School of Biology, Aristotle University of Thessaloniki, 54124 Thessaloniki, Greece; 3 ZooPlantLab, Department of Biotechnology and Biosciences, University of Milano-Bicocca, Piazza della Scienza 2, 20126 Milan, Italy; 4 Department of Geography, Ghent University, Krijgslaan 281, 9000 Ghent, Belgium

**Keywords:** Biodiversity, Molecular ecology

## Abstract

Monitoring biodiversity is of increasing importance in natural ecosystems. Metabarcoding can be used as a powerful molecular tool to complement traditional biodiversity monitoring, as total environmental DNA can be analyzed from complex samples containing DNA of different origin. The aim of this research was to demonstrate the potential of pollen DNA metabarcoding using the chloroplast *trn*L partial gene sequencing to characterize plant biodiversity. Collecting airborne biological particles with gravimetric Tauber traps in four Natura 2000 habitats within the Natural Park of Paneveggio Pale di San Martino (Italian Alps), at three-time intervals in 1 year, metabarcoding identified 68 taxa belonging to 32 local plant families. Metabarcoding could identify with finer taxonomic resolution almost all non-rare families found by conventional light microscopy concurrently applied. However, compared to microscopy quantitative results, Poaceae, Betulaceae, and Oleaceae were found to contribute to a lesser extent to the plant biodiversity and Pinaceae were more represented. Temporal changes detected by metabarcoding matched the features of each pollen season, as defined by aerobiological studies running in parallel, and spatial heterogeneity was revealed between sites. Our results showcase that pollen metabarcoding is a promising approach in detecting plant species composition which could provide support to continuous monitoring required in Natura 2000 habitats for biodiversity conservation.

## Introduction

Monitoring biodiversity in natural ecosystems, and particularly in protected areas (e.g. the European network Natura 2000), is becoming of increasing importance in ecological research to document the consequences of the recognized biodiversity loss of the last decades^1,2^. Metabarcoding provides a powerful tool for rapid surveys of plant and animal communities to acquire high-resolution information on biodiversity and its changes over long timescales and large regions^3^. Environmental DNA (eDNA) analysis, in particular, that can identify multiple species in bulk samples^4^ has the potential to provide a swift improvement in estimating community biodiversity, fostering new insights on diversity components (alpha, beta and gamma diversity) for organisms and ecosystems^5–9^.

As regards plant biodiversity, pollen richness is often utilized as a proxy to estimate floristic richness in the catchment area^10^. Traditionally, pollen assemblages are analyzed via light microscopy based on pollen morphology^11^. Specific caveats are linked to peculiar morphological features of pollen grains, which can be shared among different species, genera (e.g. genera in Poaceae family) or even families (e.g. Cupressaceae and Taxaceae ^12,13^. This limitation to taxonomic precision can be tackled by the exploitation of the DNA-based approaches which recently revolutionized environmental monitoring^14^. Pollen DNA from environmental samples has been used in recent years as a matrix in different fields, including the study of airborne allergenic pollen^12,15,16^, plant–pollinator interactions^17–19^ and provenance of honey^20,21^.

High Throughput Sequencing (HTS) enables the recovery of DNA sequence data directly from environmental samples, increasing the scalability of biodiversity surveys^22^. HTS can quickly produce a massive amount of DNA sequences with ease^23^, and increasingly accessible DNA reference databases facilitate taxonomic assignment to single (barcoding) or multiple (metabarcoding) species^24,25^. Environmental samples, such as air samples, can easily undergo degradation^26^ and therefore short markers are used ^3^. Between different short fragment markers, the chloroplast *trn*L partial gene sequencing has been previously used with airborne pollen showing to be sufficiently variable and appropriate for amplification^12,15^. Previous surveys have compared different matrices (air samples, plant material, pollen traps) to detail overlapping results between pollen morphology and DNA analyses finding a good agreement between the methods^15,16,18,27^. However, a debate is still ongoing about the effectiveness of plant metabarcoding used for the relative quantification of plant communities^21,28^, with studies supporting the quantitative potential of a multi-locus approach^29,30^.

**Figure 1:**
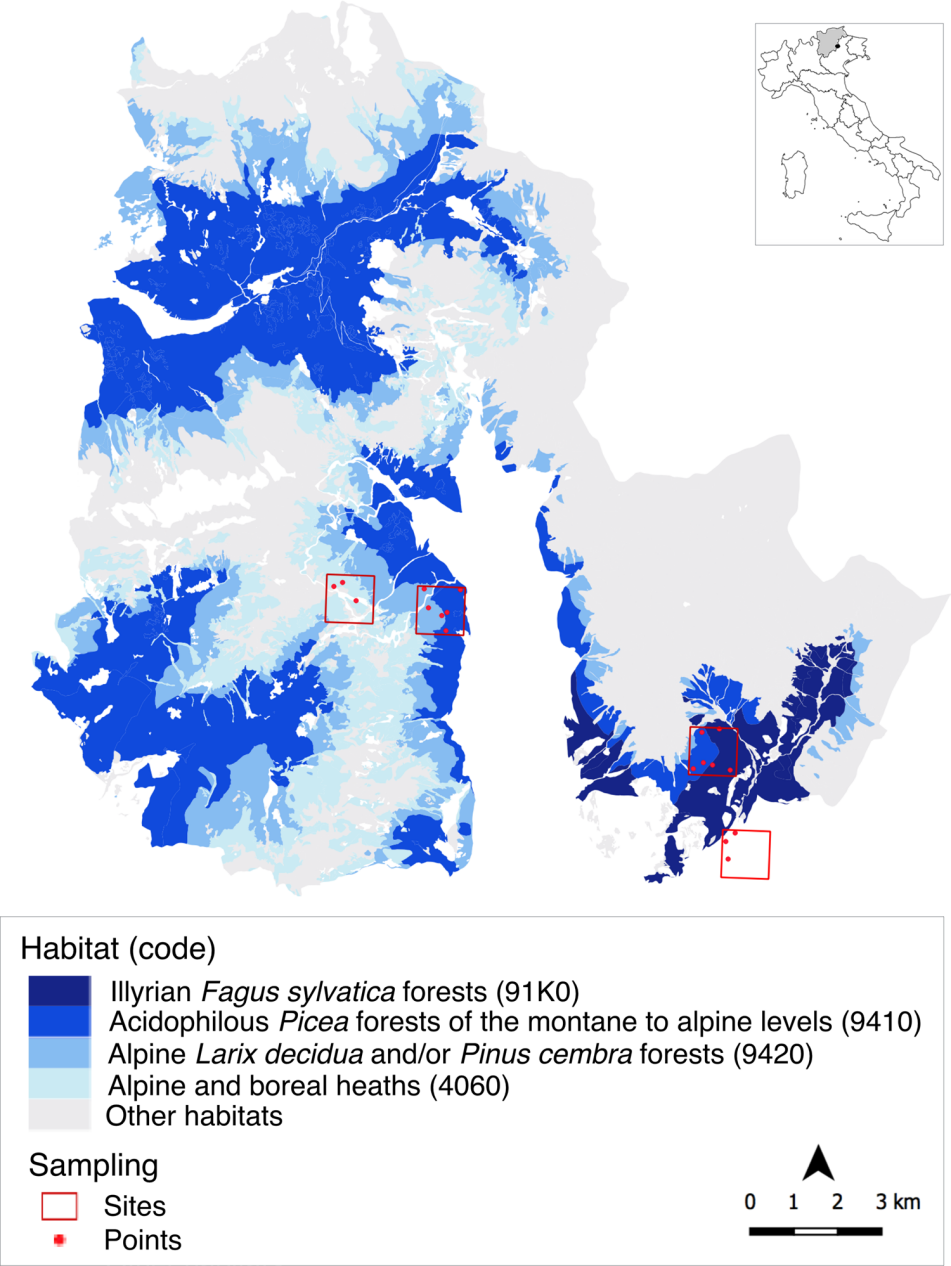
The sampling area in the National Park of Paneveggio–Pale di San Martino, Italy. The habitats in quadrats (sites) and sampling points (points) that were selected for the study are reported, as generated in QGIS3 (version 3.10.13, https://www.qgis.org).

This study, following recently optimized protocols^12^, aimed to demonstrate the potential of pollen DNA metabarcoding, using *trn*L partial gene high throughput sequencing, to characterize and monitor plant biodiversity in protected areas, (e.g. the European ecological network Natura 2000) and to be used as a tool to inform efforts related to biodiversity conservation of species and habitats in a long-term perspective. A direct comparison with the traditional methods of pollen analysis was performed by light microscopy on a subset of samples to evaluate data overlapping and complementarity. We also investigated how the DNA-based *trn*L partial gene sequencing of pollen analysis can capture spatio-temporal patterns of airborne pollen, and, hence, its potential to semi-quantitatively track vegetation changes. The study encompassed the four most prevalent EU Natura 2000 habitats within the Natural Park of Paneveggio Pale di San Martino (eastern Italian Alps), a study site selected by several conservation and monitoring projects over the last decades. In particular, each habitat (i.e. Beech forest; Spruce forest, in two quadrates; Larch forest and Alpine heath) was monitored in three sampling points (within-habitat replicates) and three non-EU sampling points adjacent to the park were also monitored to account for low-altitude vegetation (Fig. 1). Overall, the study included 18 sampling points along an altitudinal gradient (Table 1) and the sampling covered a 1-year time span: October 2014–March 2015, March–July 2015 and July–October 2015.

**Table 1:** The habitat types (according to the EU coding system) selected for the study are described.

Habitat type	Habitat code	Altitudinal range (m)	Geographical location	% cover
Land principally occupied by agriculture, with significant areas of natural vegetation/coniferous forest (Lowland)	CLC 243/CLC 312	1050–1080	Val Canali	–
Illyrian *Fagus sylvatica* forests (Beech)	91K0	1290–1460	Val Canali	3.62
Acidophilous *Picea* forests of the montane to alpine levels (Spruce low)	9410	1530–1590	Val Canali	24.31
Acidophilous *Picea* forests of the montane to alpine levels (Spruce high)	9410	1620–1760	Tognola	
Alpine *Larix decidua* and/or *Pinus cembra* forests (Larch)	9420	1780–1860	Tognola	12.82
Alpine and Boreal heaths (Alpine)	4060	2040–2180	Tognola	10.92

A contracted description of the habitats is reported in brackets.

## Results

### Biodiversity assessment through DNA metabarcoding

Our analysis detected 160 Operational Taxonomic Units (OTUs) with 12,007,712 sequenced reads, 222,370 ± 41,954 (sd) reads per sample, for a total of 54 sequenced samples. The rarefaction curves showed good sequencing effort for the samples (Supplementary Figure S1) which were rarefied to the least count among samples corresponding to 135,443 reads. Twenty OTUs, (7.2% of the total), were assigned to taxa not relevant to our work (mainly to mosses and ferns during the periods October 2014–March 2015 and July–October 2015). From the remaining OTUs, 108 (88% of the reads) were taxonomically assigned to 32 families of vascular plants (68 identified taxa) (Table 2, Supplementary Table S1) and 32 OTUs (4.8% of the reads) remained unidentified either because of low sequence identity and/or query coverage percentage or the absence of any sequence classification result, even when compared to the complete ‘Nucleotide’ Genbank database. The results of the taxonomic assignment to vascular plants are presented in Supplementary Table S1. The OTU sequences were assigned to plant taxa with at least 95% identity and coverage, from which 70% of the OTUs had ≥ 98% sequence identity with the assigned taxa. The positive control of the DNA extraction, *Corylus avellana* pollen, was correctly identified after HTS*.* From the 19 negative controls included in the extraction plate, one negative control was selected for sequencing, the only one with sufficient amplicon concentration (2 ng μl^−1^). In this sample two OTUs were detected (263,649 reads), both assigned to *Quercus* spp. and contributing < 0.005% to the counts of the rest of the samples. The average amplicon concentration of the three pooled PCR products (before library preparation) was 8.7 ± 4.6 (sd) ng μl^−1^.

**Table 2:** Quantitative data for the sequenced reads and the vascular plant taxa that were identified at the different sampling periods and habitats.

	No. of OTUs (sequenced reads)	No. of identified taxa	No. of identified families
**Sampling period**			
October 2014–March 2015	142 (3,744,854)	64	30
March–July 2015	135 (4,182,915)	64	31
July–October 2015	109 (4,079,943)	46	21
**Habitat type**			
Lowland	128 (2,225,089)	63	29
Beech forest	129 (1,744,374)	61	30
Spruce forest (low)	124 (2,195,166)	58	28
Spruce forest (high)	129 (2,109,992)	55	24
Larch forest	113 (1,851,947)	52	22
Alpine heath	130 (1,881,144)	53	24
Total	160 (12,007,712)	68	32

The full names of the corresponding habitats are given in this table.

The sampling period July–October 2015 had a relatively lower number of OTUs and identified vascular plant taxa compared to the other periods (March–July 2015 and October 2014–March 2015). When grouped by habitat, the lowest number of OTUs and assigned taxa were found in Larch forests (Table 2). The alpha diversity of the habitats in each sampling period estimated as the number of OTUs per sample, is presented in Supplementary Figure S2. The interaction between sampling period and habitat category significantly influenced the alpha diversity (ANOVA, *p* < 0.01). Specifically, OTU richness decreased in July–October 2015 for all habitats, while in October 2014–March 2015 Larch forests and Alpine heath showed significantly lower plant biodiversity than Spruce, Beech and Lowland habitats.

**Figure 2:**
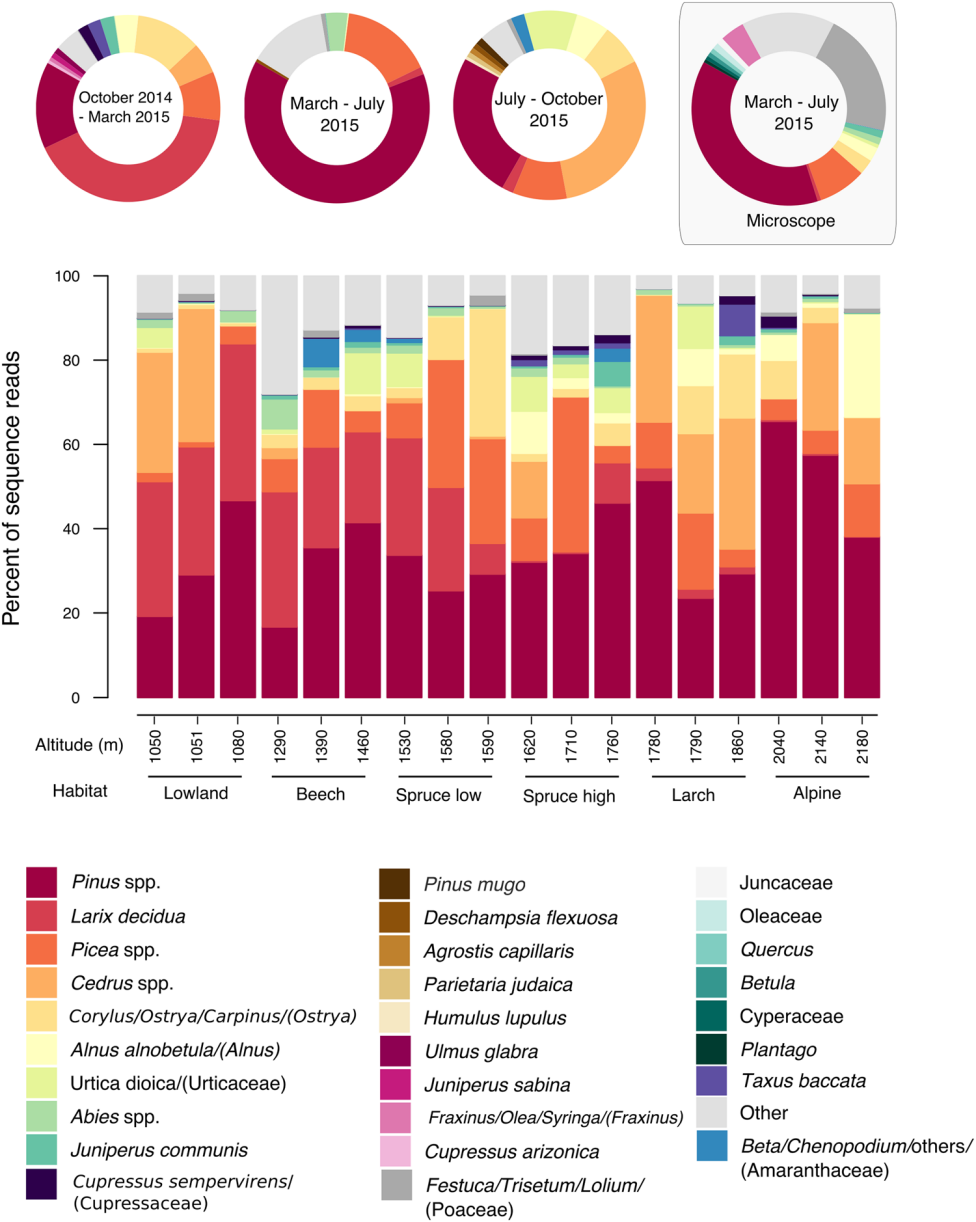
Taxa summary plots: Doughnut pie chart for all three sampling periods as derived by HTS and for the period March–July 2015, as derived by microscopy (up) and barplot for each sampling point (ordered in the x-axis from low to high altitudes) as derived by HTS. The charts represent relative abundance of sequences reads and microscope counts (≥ 0.5% of each period’s total for the doughnut plots and ≥ 0.5% of the annual total for the barplot). All the rest of the taxa are grouped under ‘Other' taxa. If the level of taxonomic identification differed between methods, given in parenthesis is the level after the microscopic method. For the full plant taxa assignment data see Supplementary Table S1. The full names of the corresponding habitats are given in Table 1.

Of the 68 identified taxa from the HTS dataset, 42 were woody (trees and shrubs) and 26 herbaceous, 10 of which were graminoids (Supplementary Table S1). Fifteen of these taxa were not present in the plant checklist of the park^31^ (Supplementary Table S1). There were 13 main pollen taxa contributing at least 0.5% to the total number of the sequence reads (Supplementary Table S1). These were *Pinus* spp. (36.8%), *Larix decidua* (14.4%), *Cedrus* spp. (12.4%), *Picea* spp. (11.6%), *Corylus/Ostrya/Carpinus* (5%), *Alnus alnobetula* (2.9%), *Urtica dioica* (2.8%), *Abies* spp. (1.5%), *Juniperus communis* (0.7%), *Beta/Chenopodium/*others (0.6%), *Taxus baccata* (0.6%), *Festuca/Trisetum/Lolium* (0.5%) and *Cupressus sempervirens* (0.5%). The relative abundance of the most represented taxa (with a relative contribution at least 0.5% to the sequenced reads (in each period and in total) is presented in Fig. 2. Pinaceae taxa (*Pinus, Picea, Cedrus, Abies*) were dominant in all three periods, Betulaceae *(Corylus/Ostrya/Carpinus, Alnus, Betula*), Cupressaceae (*Juniperus, Cupressus*) and Taxaceae (*Taxus*) were also abundant in the period October 2014–March 2015 as well as Amaranthaceae (*Beta/Chenopodium*/others), Betulaceae (*Corylus/Ostrya/Carpinus, Alnus, Betula*), and Urticaceae (*Urtica/Parietaria*) in the period July–October 2015. *Pinus* spp. were the most represented plant taxa in all habitats for the entire 1-year dataset. As regards the other taxa, each habitat showed a distinct plant biodiversity spectrum, while *Picea* spp. were mostly found in the samples of the Spruce forests (Fig. 2).

**Figure 3:**
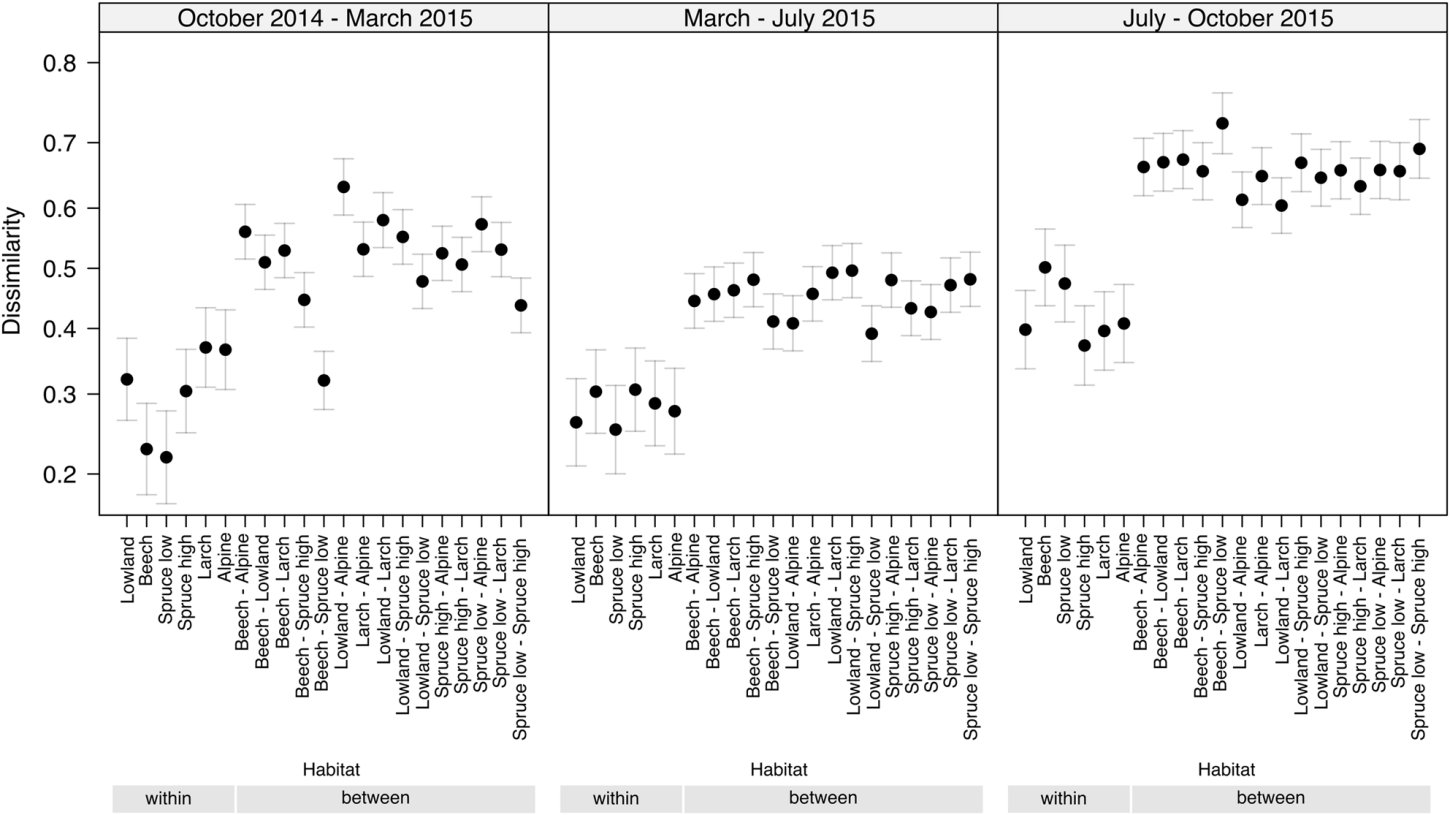
Effect plots showing the differences between the habitat and period categories included in the Generalized Linear Model (quasi-binomial error distribution and logit link function) formulated with pairwise Jaccard dissimilarity index values (mean ± standard error) The full names of the corresponding habitats are given in Table 1.

Beta diversity as calculated by Jaccard dissimilarity between samples is presented within and between habitats (Table S4, Fig. 3). Jaccard dissimilarity values were lower within habitats than between habitats for all three periods. For the within-habitat dissimilarity the period March–July 2015 had the highest values. Beta diversity was affected by the sampling period (PERMANOVA, *p* < 0.001, pseudo-*F* = 11.1, *R*^2^ = 0.30) and the habitat (PERMANOVA, *p* < 0.01, pseudo-*F* = 1.5, *R*^2^ = 0.39). According to the Generalized Linear Model predicting the Jaccard dissimilarity values as function of sampling period, habitat and their interaction (categorical variables), significant differences in beta diversity of different periods and habitats were detected, while also the interaction term of the latter variables had a significant effect on the variation of species composition (Supplementary Table S3). The effect of habitat (categorical variable) to Jaccard dissimilarity values is displayed in Fig. 3 showing higher dissimilarity between habitats than within habitats.

### Comparisons between DNA metabarcoding and microscopic analysis

The period March–July 2015, which is the main pollen season for most of the vascular plants of the study, was selected for microscopic analysis. The number of identified taxa is presented in Supplementary Table S4 for each habitat. The mean number in the habitats was 23 ± 4 (sd) taxa, while in total we could identify 50 taxa. The lowest number of identified taxa was recorded in Beech and Spruce high habitats, while for the same period, HTS recorded the lowest numbers in Spruce high and Larch forests. The most represented ones were *Pinus* spp. (37.9%), Poaceae (20.9%) and *Picea* spp. (8%) and 19 taxa contributed > 0.5% to the counts (Fig. 2, Supplementary Table S1). Beta diversity, as estimated by Jaccard distance (Supplementary Figure S4), showed to be affected by the habitat type (PERMANOVA, *p* < 0.05, pseudo-*F* = 1.45, R^2^ = 0.37).

**Figure 4:**
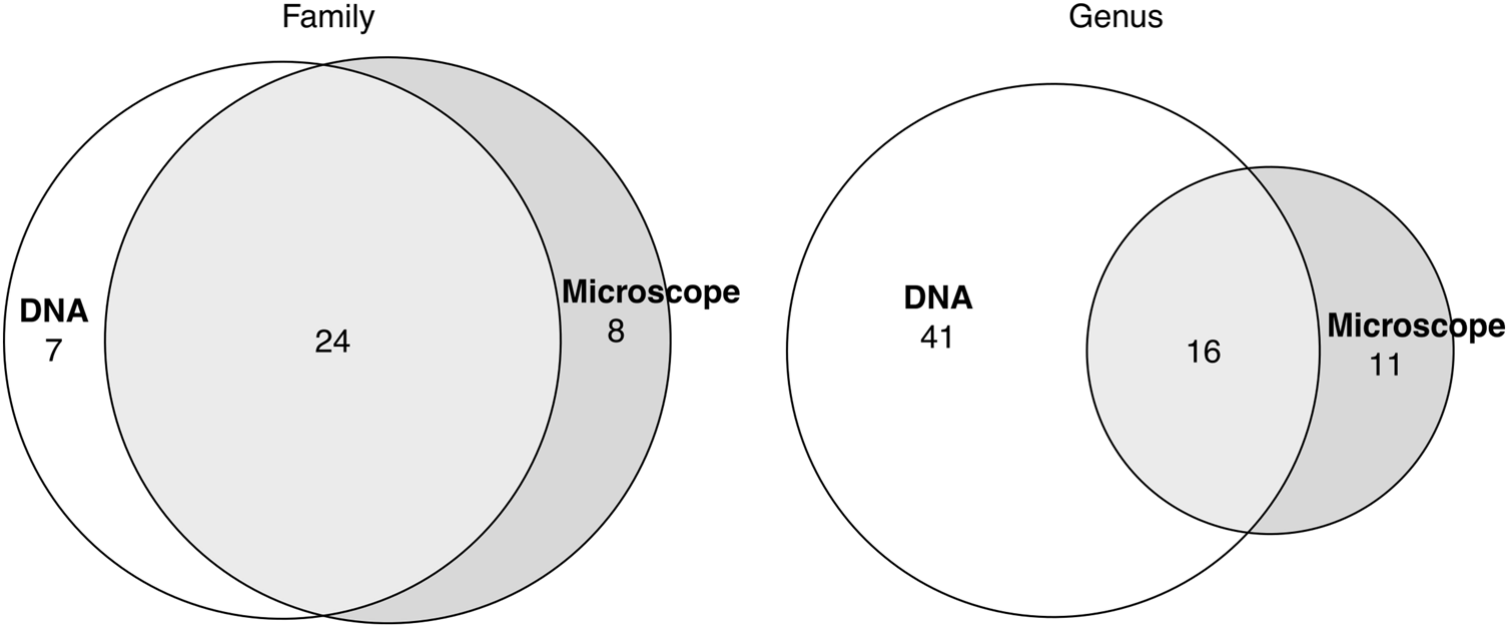
Venn diagrams with the number of families found by DNA metabarcoding and microscope and the common genera from those families as detected during March–July 2015.

In total, DNA metabarcoding detected 68 plant taxa compared to 50 revealed by microscope (Supplementary Table S1). We identified a total of 39 families, 24 of which were identified by both methods (Fig. 4). Within these common families, DNA metabarcoding could distinguish 57 genera or groups of genera compared to 27 identified by the microscopic method (Fig. 4). In general, families not shown in the outputs of one of the two methods (eight and seven absent from the DNA metabarcoding and microscope outputs, respectively) were scarcely represented also by the other. Cyperaceae and Polygonaceae, although found with considerable abundance with conventional light microscopy analysis (0.4% and 0.6%, respectively), did not appear in the HTS results (Supplementary Table S1).

**Figure 5:**
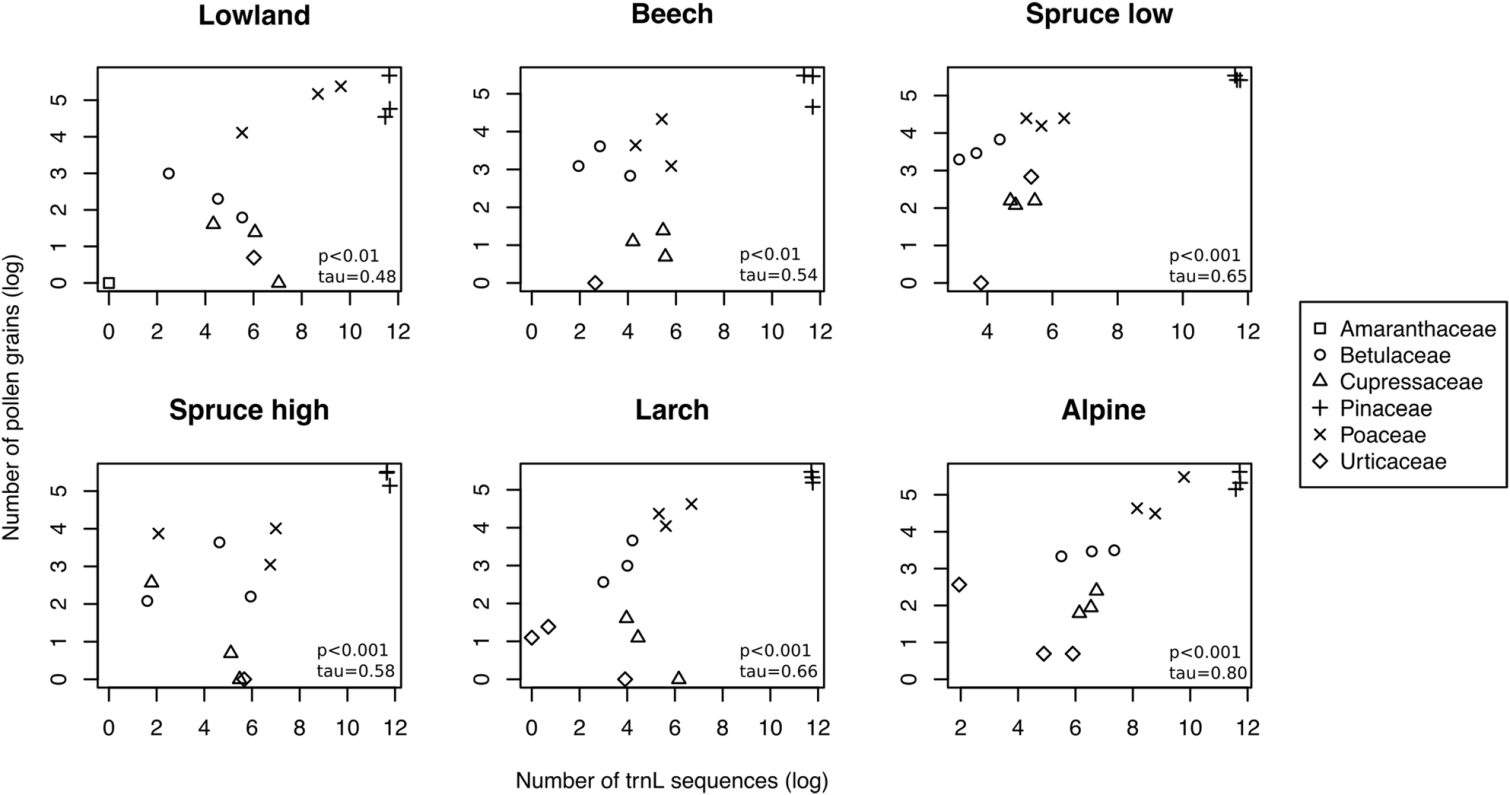
Log-scaled quantification results as estimated by *trn*L metabarcoding counts (x-axis) and pollen microscope counts (y-axis). The results are summarized for the top five families identified by metabarcoding by habitat group for the period March–July 2015. *P*-values and the correlation coefficient from Kendall tau rank correlation tests are provided in the plot of each habitat. The full names of the corresponding habitats are given in Table 1.

Log-scaled quantification results of pollen grains (microscope) and *trn*L sequence reads (metabarcoding) were compared between the two methods for the samples of March–July 2015 for each habitat within the altitudinal gradient (Fig. 5). For all habitats, there was significant correlation, i.e. *p* < 0.01 for Lowland (tau = 0.48) and Beech forest (tau = 0.54); *p* < 0.001 for Spruce low (tau = 0.59), Spruce high (tau = 0.67), Larch forest (tau = 0.67) and Alpine heath (tau = 0.8), which showed the highest correlation coefficient. Comparing the contribution of each family to the pollen spectra of all habitats, as derived by the two methods, we observe that the microscopic method systematically detected a higher frequency of Poaceae, Betulaceae and Oleaceae pollen than metabarcoding, whereas the latter showed a higher frequency of Pinaceae pollen (Fig. 2, Supplementary Table S1). For both DNA metabarcoding and pollen counts, *Pinus* was the most abundantly represented taxon (Supplementary Table S1). Notably, *Fagus* was not present in the HTS results and scarcely represented by the microscopic method. 11.7% of pollen grains were unidentified by the microscopic analysis due to unclear morphology, resulting mainly from the degradation of the pollen cell wall structure. 10.4% of sequence reads were unidentified (no significant match with reference sequences) by metabarcoding in the same period.

Procrustes analysis revealed that there was no significant accordance between distance matrices generated by HTS and microscopic analysis for the period March–July 2015, based on the permutational test (no statistical significance of the Procrustean fit, correlation 0.4, 999 permutations) (Supplementary Figure S4).

### Spatio-temporal patterns of pollen taxa

For each sampling period, the results of the HTS data for the identified plant taxa are presented in Supplementary Table S1. The main HTS pollen taxa contributing with at least 0.5% in the total reads of each period are *Larix decidua*, *Pinus* spp.*, Corylus/Ostrya/Carpinus*, *Picea* spp.*, Cedrus* spp.*, Alnus alnobetula*, Cupressaceae (*Juniperus communis*, *Cupressus sempervirens, Juniperus sabina), Taxus baccata*, *Ulmus glabra* and *Fraxinus/Olea/Syringa* for the period October 2014–March 2015; *Pinus* spp., *Picea* spp.*, Abies* spp.*, Larix decidua and Festuca/Trisetum/Lolium* for March–July 2015; and *Cedrus* spp., *Pinus* spp.*, Picea* spp.*,* Urticaceae (*Urtica dioica* and *Parietaria judaica*), *Corylus/Ostrya/Carpinus, Alnus alnobetula, Larix decidua, Beta/Chenopodium/*others and Poaceae *(Agrostis capillaris, Festuca/Trisetum/Lolium, Deschampsia flexuosa)* for July–October 2015. Significantly higher occurrence according to ANOVA was recorded for *Cupressus sempervirens* (*p* < 0.001), *Juniperus communis* (*p* < 0.01) and *Larix decidua* (*p* < 0.001) in the period October 2014–March 2015, for *Pinus* spp. (*p* < 0.001) and *Abies* spp. (*p* < 0.001) in the period March–July 2015 and for *Urtica dioica* (*p* < 0.001), *Cedrus* spp. (*p* < 0.01) and *Beta/Chenopodium/*others (*p* < 0.05) in the period July–October 2015.

For the 13 main plant taxa, detailed quantitative HTS and microscope data are presented for each habitat during the period March–July 2015 (Supplementary Table S2). Significant spatial patterns were revealed according to ANOVA for *Larix decidua* with the highest occurrence recorded in the Larch forest (*p* < 0.05), *Alnus alnobetula* with the highest occurrence in the Alpine heath (*p* < 0.05) and *Festuca/Trisetum/Lolium* with the highest occurrence in the Lowland and Alpine heath (*p* < 0.05) (Supplementary Table S2). Similarly, for the microscope data spatial patterns were statistically significant for *Larix,* with the highest occurrence recorded in the Larch forest (*p* < 0.001)*,* for *Alnus,* with the highest occurrence in the Alpine heath (*p* < 0.05), and for *Cedrus* with the highest occurrence in the low Spruce forest (*p* < 0.05). Occurrence of *Picea* was higher in the Spruce and Larch forests and for Poaceae in the Lowland and Alpine habitats, but in both cases differences were not significant, (*p* < 0.05) (Supplementary Table S2)*.*

According to metabarcoding results, high read numbers of the main plant taxa were recorded when high pollen counts were also detected in airborne pollen data from the aerobiological station of the Park (Supplementary Figure S3). Exceptions are *Corylus/Ostrya/Carpinus*, *Alnus alnobetula* and *Pinus* spp., for which eDNA showed that their pollen was at relatively high concentrations both during their pollen seasons and at different times. In contrast, for *Beta/Chenopodium/*others and *Urtica dioica*, plant DNA was detected only in the period July–October 2015, although their pollen season was longer, starting within the period March–July 2015 (Supplementary Figure S3).

The majority of the main pollen taxa (11 of 13 taxa) have representatives in at least one of the habitat types examined^31^, except for *Cupressus sempervirens* and *Cedrus* spp. (Supplementary Figure S3) that do not occur within the Park and are probably transported from surrounding areas; notably, *Cedrus* is found at all altitudes of all periods. Also *Pinus* spp.*, Larix decidua* and *Picea* spp. were retrieved from all samples (Supplementary Table S1).

## Discussion

In this study, we characterized airborne pollen patterns by DNA metabarcoding within the most represented Natura 2000 habitats in Paneveggio Pale di San Martino Natural Park (North Italy), using eDNA from air samples. Chloroplast *trn*L partial gene sequencing provided an informative list of plant taxa growing along an altitudinal gradient of the Italian Alps with a finer taxonomic resolution when compared to the conventional light microscopy. It also uncovered spatio-temporal pollen patterns. Most of the taxa detected with at least 0.5% of total reads by DNA metabarcoding are represented in the vegetation of the park, and they were mostly recorded at the sampling times corresponding to their pollen season, as confirmed by the aerobiological sampling. This result is particularly useful for biodiversity assessments and monitoring in the present study area and also suggests that the method has the potential to be applied successfully in other areas.

Due to various factors that can influence quantitative results of metabarcoding, we applied semi-quantitative indices for our community analysis with HTS data: OTUs richness to estimate alpha diversity and the presence/absence-based Jaccard index as a measure of beta diversity^32^. Overall, alpha diversity for the habitats of lower altitude (1050–1760 m), i.e. Beech and Spruce forest was higher and revealed more vascular plant taxa with a total of 30 families when compared to habitats at higher altitudes (1780–2180 m), i.e. the Larch forest and Alpine Heath, with a total of 24 families (Supplementary Figure S2, Table 2). This suggests that a pollen metabarcoding approach can detect known differences in taxa richness along elevational gradients in mountainous areas^33^. In addition, the Jaccard dissimilarity of taxa within samples of the same period was significantly lower than between periods (PERMANOVA, *p* < 0.001, pseudo-*F* = 11.1, *R*^2^ = 0.30), showing that the sampling period significantly influences beta diversity. This is an important finding because it shows that pollen metabarcoding can detect changes with time and, hence, that it can be used for monitoring purposes. Beta diversity, estimated with Jaccard index, showed that the habitat replicates efficiently represented the different habitats by both conventional (PERMANOVA, *p* < 0.05, pseudo-*F* = 1.45, R^2^ = 0.37) and metabarcoding (PERMANOVA, *p* < 0.05, pseudo-*F* = 1.45, R^2^ = 0.37), since the variation in species composition was lower within than between habitats. Additionally, the Generalized Linear Model (quasi-binomial error distribution and logit link function) predicting the Jaccard dissimilarity values as function of sampling period, habitat and of their interaction term showed significant differences in the pairwise comparisons between habitats (Fig. 3, Supplementary Table S3).

When comparing qualitatively, in terms of identified taxa, conventional light microscopy and *trn*L partial gene sequencing, results showed good agreement, since 61% of the families were represented by both methods. For plant families detected by just one of the two methods, pollen abundance based on read counts or microscope counts was very low (Supplementary Table S1). Two families, Cyperaceae and Polygonaceae, were not detected by DNA metabarcoding, although considerably abundant in the light microscopy results. However, Cyperaceae is a family known to have low detection with the *trn*L barcode^34^. The result of not detecting specific taxa by this procedure could be due to primer mismatching during PCR and lack of amplification with a consequent exclusion from the sequencing results^15,35^ Other PCR biases such as differences in GC content^36^ or differences in cpDNA copy numbers^12^ may also affect the detection of specific taxa. To reduce PCR bias resulting from primer mismatching, an optimization of the PCR protocol or the use of a combination of primer pairs^30^ could be applied to improve sensitivity towards the plant species of interest.

Within the common families, the taxonomic information resulting from the metabarcoding method was richer (27% more taxa) and provided a finer resolution (53% more genera) when compared to the light microscopy results. The observed improvement in taxonomic information is in agreement with previous studies^15,19,20,27^. In particular, for some taxa that are usually identified only collectively (at family level or higher) with conventional light microscopy (e.g. grasses, for which pollen grains have common morphological features), the targeted HTS led to the identification of different genera^13^. This is highly relevant for biodiversity estimates, as well as for health issues linked to pollen allergy, e.g. more precise diagnosis and treatment of human sensitivity to aeroallergens^37^.

Nevertheless, quantitative estimates (abundance) of the plant biodiversity showed some notable differences between the DNA and microscope-based methods. When comparing the results of the two methods by habitat, Alpine heaths showed better correlation of relative abundance of reads and pollen grains (tau = 0.8, *p* < 0.001) compared to forested zones. This might also have led to overall low correlation generated by Procrustes analysis when comparing the ordinations generated by the two methods. In addition, when focusing in the most abundant taxa (at least 0.5% of contribution to the HTS counts), there were taxa that showed similar peaks in abundance compared to microscope results, such as *Larix* in Larch forest, *Picea* in Spruce forest, Poaceae and *Alnus* in the Alpine heaths.

For the main pollen season that we used for comparisons, although *Pinus* was the most abundantly represented taxon by both methods, Pinaceae were much more represented by DNA metabarcoding. In contrast, light microscopy identified more Poaceae, Betulaceae and Oleaceae. Similar differences were reported by other authors when either nuclear or chloroplast markers were used to analyze mixed pollen samples^15,20,21,27^. This quantitative issue could be linked to the amplification biases inherent in the PCR methodological step that can possibly lead to overamplification of some templates^38,39^. Other studies have applied capture enrichment which led to more accurate results avoiding PCR biases^40^. In addition, pollen structure can lead to over and under-representation of some taxa as demonstrated by previous studies^41^. Studies investigating predefined mock communities have attempted to give insight to quantitative issues^42–44^, but more controlled experiments with detailed knowledge of the eDNA ecology and simulations in all ecosystems would be needed to correlate the observed with the true abundance^42^.

In several cases, plant taxa were found in samples collected from sites where the producing plants were absent. This was the case for *Taxus baccata*, *Beta/Chenopodium/*others, *Corylus/Ostrya/Carpinus*, *Abies* spp. and *Pinus* spp. that grow in other sites inside the Park, but also for *Cedrus* spp. and *Cupressus sempervirens* that grow outside it. All Pinaceae taxa (*Pinus* spp.*, Larix* spp.*, Cedrus* spp.*, Picea* spp.*, Abies* spp.) were present in all seasons and almost all habitats. Since those taxa were in high quantity and they were not amplified in negative controls, we do not consider them as contaminants of the process. This finding can be partly explained by transfer from elsewhere^45^, but not only, since these plant taxa were detected at periods that do not correspond to their pollen seasons. For Pinaceae, this could be explained as the result of the huge amount of pollen produced locally, which may remain on the ground for a long time, gets resuspended by the wind and can be found in near ground-level samples even in periods when it is not produced and released by the plants. Such relic DNA has been detected and quantified in water and soil studies^46–48^; this study provides initial evidence that it can also be detected in air samples. *Pinus* was the most abundant plant/pollen taxon after either method, thanks to its very light pollen, easily transported by the wind^49^, more than that of other Pinaceae taxa. Van der Knaap^50^, using Tauber traps, found higher amounts of *Pinus* pollen above the treeline than in lower mountainous areas, indicating upslope transport by wind currents. Notably, the absence of *Fagus* (a prevalent tree in the Park) from the DNA metabarcoding results agreed with the aerobiological monitoring data of the Paneveggio Pale di San Martino Natural Park. Given the natural variability in the reproductive output of beech in different years^51^, this may be due to a much lower pollen production of beech in the year of study.

Overall, pollen metabarcoding increased considerably our ability to identify pollen^52^. Within the ecological network Natura 2000, eDNA analysis could be used as an efficient tool to characterize plant biodiversity and monitor diversity changes over space and time. Thanks to the fine taxonomic assignment, the method could be used in plant management, for instance to detect potential invasion of non-native species; this could be the case for ragweed pollen which can affect human health with its high allergenicity^53^. To resolve quantitative issues, more effective techniques could be applied in the future, such as complementing with other or new target barcodes, which would also give better resolution for some plant families^29^. At the same time, more extensive field experiments in different natural ecosystems could be planned at different time scales to examine the accuracy of the quantitative information^42^. DNA metabarcoding has therefore the potential to be applied successfully in other areas for biodiversity management and/or conservation purposes to ensure the protection of plant biodiversity and habitats in a long-term perspective.

## Materials and methods

### Sampling design

The sampling areas in the Natural Park of Paneveggio Pale di San Martino belong to the San Martino chain (Tognola mountain) in the west and the Val Canali chain in the southeast. The four most prevalent habitats^54^, covering 52% of the Park’s surface, were selected (among a total of 12 Natura 2000 habitats that cover > 90% of the area): Spruce forests (code 9410: Acidophilous *Picea* forests of the montane to alpine levels; 24.3%) in low and high altitudes, Larch forests (code 9420: Alpine *Larix decidua* and/or *Pinus cembra* forests; 12.8%), Alpine habitat (code 4060: Alpine and boreal heaths; 10.9%) and Beech forests (code 91K0: Illyrian *Fagus sylvatica* forests; 3.6%). An area outside of the Park was selected to account for lower altitude biodiversity, classified according to Corine Land Cover classification system (CLC) as Land principally occupied by agriculture, with significant areas of Natural vegetation (CLC 243; two points) and coniferous forest (CLC 312; one point). The study included 18 sampling points along the altitudinal gradient of the park. Three points were selected as replicates to account for within-habitat variability of the plant biodiversity. The sampling points were located randomly in quadrats (1 km × 1 km) of east exposure, with each quadrat spanning one or two different habitats. Spruce forest was replicated in the two different quadrats, to account for the variability related to geographic location (Table 1, Fig. 1).

A total of 18 sampling points were equipped with gravimetric Tauber traps, and pollen samples were collected over three different periods covering a 1-year time span: October 2014–March 2015, March–July 2015 and July–October 2015. At each sampling point two Tauber traps were positioned at the same pole, further used as site replicates for HTS and microscope analysis.

### Tauber traps, sample processing and microscopic analysis

Each Tauber trap had 700 mL of a preservative solution (1:1:1 water, alcohol, glycerol, plus 2 g L^−1^ phenol)^12^. All traps were installed at the different sampling points close to the ground (half meter), tied to poles. A collar was placed on the apertures of the traps that was covered with a 5 mm mesh net to prevent collection of larger particles^49^. Airborne particles were collected by gravitational settling, and sampling solutions were filtered and processed following the protocol of Leontidou et al.^12^. The obtained pellets were the starting material for microscopic and molecular analysis and they are considered biological replicates, since they derived from exactly the same area after following the same sampling and processing methodology.

The period of March–July 2015 was selected for comparisons between metabarcoding and microscopic analysis, since it is the main pollen season for most of the plant families in the study area according to aerobiological data. Samples for the microscopic analysis (18 out of a total of 54) were processed by smearing a small aliquot of the pellet on a slide and coloring with basic fuchsin. Morphological pollen analysis was performed using an optical microscope (Leitz Diaplan, Ernst Leitz Wetzlar, Wetzlar, Germany) under 400× magnification. Approximately 400 pollen grains were analyzed per each sample. A local pollen reference collection was used as a quality check for pollen identification by light microscopy^55,56^.

### Vegetation and aerobiological data

For vegetation data, we used the plant checklist of the Park^31^. Aerobiological data including the concentrations of pollen taxa per cubic meter of air were retrieved from the archive of the monitoring station in Villa Welsperg, the visitor’s centre of the Park (Val Canali, 46°11′57.3″N 11°52′06.3″E, 1050 m). The station is equipped with a Lanzoni volumetric sampler (VPSS, Lanzoni, Bologna, Italy) and pollen analysis is performed by light microscopy, following the European standard (UNI CEN/TS 16868:2015).

### DNA extraction and sequencing

Pollen pellets originating from 54 Tauber traps were used as starting material for DNA extraction using Nucleomag kit (Macherey–Nagel, Düren, Germany) and the Kingfisher (Thermo Fisher Scientific, Waltham, MA, USA) automated DNA extraction system. In particular, with the use of distilled water, 200 μl were transferred from the initial pollen pellet sample to the extraction tube. To decrease lab contaminants, the tubes were transferred in a non-invasive DNA extraction room (separated from PCR or post-PCR rooms), where the lysis step and the preparation of the extraction plates were performed in a specialized safety cabinet. For the lysis, a step that is prone to lead to differences in DNA release between species^15^, we followed optimized protocols to increase DNA yield. In particular, one sterilized steel bead, lysis buffer and RNase were added in each tube, according to the manufacturer’s protocol and a high-energy agitation (Retsch MM200 mixer mill, Germany) was applied (30 Hz for 1 min, two steps) to mechanically disrupt cell walls. The final product was transferred in an extraction plate, where the rest of the reagents were added. We included a sample of a known species (*Corylus avellana*) as positive control and we had 19 negative controls in different stages of the extraction (14 with lysis buffer and post-lysis reagents and five wells of the plate filled only with the post-lysis reagents). The extracted DNA was eluted in 100 μl.

The eDNA was amplified with the chloroplast *trn*L primers c-A49325 and h-B49466 [5′-CGAAATCGGTAGACGCTACG-3′ and 5′-CCATTGAGTCTCTGCACCTATC-3′] (Sigma-Aldrich, Milan, Italy), with expected amplicon size 141 bp^57^. We followed the GoTaq protocol (Promega, Madison, WI, USA) in three separate reactions for each sample, to maximize the chance of detecting all species in our pollen pellets^58^. The DNA amplification was carried out in a final volume of 50 μl using 5 μl of extracted DNA. The amplification mixture contained 1.25 U GoTaq Polymerase, 1× of GoTaq Flexi Buffer, 3 mM of MgCl_2_, 0.2 mM of dNTPs and 0.2 μM of each primer. All PCR amplifications were carried out on a Veriti 96 well thermal cycler (Applied Biosystems, Foster City, CA, USA) with the following program: 2 min at 95 °C and 40 cycles of 15 s at 95 °C, 15 s at 52 °C and 30 s at 72 °C, followed by 5 min at 72 °C. The PCR products were checked using electrophoresis in Qiaxcel (Qiagen, GmbH, Hilden, Germany). Three PCR reaction products were pooled together in equal amounts. They were further gel purified using the MinElute Gel Purification kit (Qiagen) and eluted in 53 μl. The final PCR product was quantified with Qubit 2.0 Fluorometer (Life Technologies, Thermo Fisher Scientific).

Around 100 ng of the purified PCR product were used for library preparation. A total of 56 independent libraries were generated using TruSeq DNA sample preparation kit V2 (Illumina Inc., San Diego, CA, USA), pooled in equimolar ratio and then sequenced using MiSeq Reagent Kit v3 on an Illumina MiSeq platform.

### Bioinformatics processing and taxonomic assignment

Raw Illumina reads were paired and pre-processed using VSEARCH v2.5.0 ‘merge pairs’ algorithm^59^. Reads were filtered out if ambiguous bases were detected and if the read lengths were less than 100 bp. Furthermore, an expected error (= 1) was used as an indicator of read accuracy. Pollen OTUs were obtained using the ‘cluster_fast’ algorithm with a 97% sequence identity and a depth of at least 100× reads for each cluster. The centroid of the resulting cluster was chosen as the representative sequence for the taxonomic assignment step. To decrease the false positive rate in the sequence population, a chimera detection analysis was performed on the obtained reference sequences. Since there is no reference database for the *trn*L gene for chimera detection, we used the ‘uchime_denovo' algorithm that carries out a de novo analysis without a reference.

Taxonomic assignments were performed by BLASTN+ software of ncbi-blast-2.2.31+^60^. Reference OTU sequences were aligned against our local reference database, constructed to include the most widespread anemophilous taxa of the study area (Trentino)^12^. Briefly, 1470 species (46 families) known to occur in the study area were searched in the database ‘Nucleotide’ of Genbank and 1188 sequences (403 species of 46 families) were retrieved and stored in a local database as described in Leontidou et al.^12^. The e-value parameter was set to a maximum value of 1e−40, above which the assignment was not considered valid. We also asked for sequence similarity and query coverage > 95%. An additional comparison with the non-redundant nucleotide NCBI database was performed with the same parameters to check the OTUs that were not assigned by our local reference database and search for contaminants or species not related to our study (e.g. ferns).

### Statistical analysis

Diversity was analyzed with the R packages ‘vegan’^61^ and ‘phyloseq’^62^. First, a normalization of the read counts was performed to achieve equal sequencing depth among samples (rarefaction). In particular, the OTU table was rarefied (R vegan function ‘rarefy’) to the least number of sequence reads among samples and rarefaction curves were constructed (R vegan function ‘rarecurve’) to estimate the sufficiency of sequencing depth per sample. The normalized counts were used for further analysis.

Alpha diversity was estimated by calculating OTUs number in each sample (Richness). We also examined whether alpha diversity was affected by the sampling period and habitat using ANOVA tests.

To assess the effectiveness of *trn*L metabarcoding for pollen quantification we investigated the correlation between the number of *trn*L sequence reads and the number of pollen grains (microscope) for the period March–July 2015. Correlation coefficients and *p*-values were calculated using Kendall tau rank correlation tests.

As a measure of beta diversity, i.e. variation in species composition between samples, we used the presence/absence-based Jaccard pairwise dissimilarity index. Permutational multivariate analysis of variance (PERMANOVA) was used with a balanced design to estimate the significance of the categorical variables (habitat, sampling period) to the dissimilarity (R vegan function ‘adonis’). Then, we formulated a Generalized Linear Model (quasi-binomial error distribution and logit link function) predicting the Jaccard dissimilarity values as function of sampling period, habitat and of their interaction term to further elucidate the effect of the aforementioned variables including difference within and between groups. To display the effects of the independent variables to the dissimilarity values (dependent variable) we used the R package ‘effects’^63^.

To compare the communities identified by microscope and metabarcoding we performed Non-metric Multidimensional Scaling (NMDS) using the beta diversity (Jaccard) matrix and further applied Procrustes analysis on these ordinations.

Finally, we applied ANOVA tests to detect significant differences in the main contributing pollen taxa in different sampling periods and habitats.

## Supplementary Information

Supplementary Information 1.

Supplementary Information 2.

Supplementary Information 3.

Supplementary Information 4.

Supplementary Information 5.

## Data Availability

The datasets generated and/or analysed during the current study are shared in figshare [10.6084/m9.figshare.12789950]. Raw reads were submitted to the European Nucleotide Archive (ΕΝΑ) under the Accession Number PRJEB40729.
