# Supplementary material for: Plant biodiversity assessment through pollen DNA metabarcoding in Natura 2000 habitats (Italian Alps)
Source: Sci Rep. 2021 Sep 14;11:18226. doi: 10.1038/s41598-021-97619-3 (PMC8440677; doi:10.1038/s41598-021-97619-3)
Supplement: Supplementary file 5 — Supplementary Information 5. [file 41598_2021_97619_MOESM5_ESM.pdf]

Plant biodiversity assessment through pollen DNA metabarcoding in Natura 2000 habitats (Italian Alps)

Kleopatra Leontidou[1,2]*, Despoina Vokou[2], Anna Sandionigi[3], Antonia Bruno[3], Maria Lazarina[2], Johannes De Groeve[1,4], Mingai Li[1], Claudio Varotto[1], Matteo Girardi[1], Maurizio Casiraghi[3], Antonella Cristofori[1]

[1]*Biodiversity and Molecular Ecology Dept., Research and Innovation Centre, Fondazione Edmund Mach, Via E. Mach 1, 38010 San Michele all' Adige, Trentino, Italy*

[2]*Department of Ecology, School of Biology, Aristotle University of Thessaloniki, 54124 Thessaloniki, Greece*

[3]*ZooPlantLab, Department of Biotechnology and Biosciences, University of Milano-Bicocca, Piazza della Scienza 2, 20126, Milan, Italy*

[4]*Department of Geography, Ghent University, Krijgslaan 281, 9000 Ghent, Belgium*

*Corresponding author: Kleopatra Leontidou, cleopatra.leontidou@gmail.com

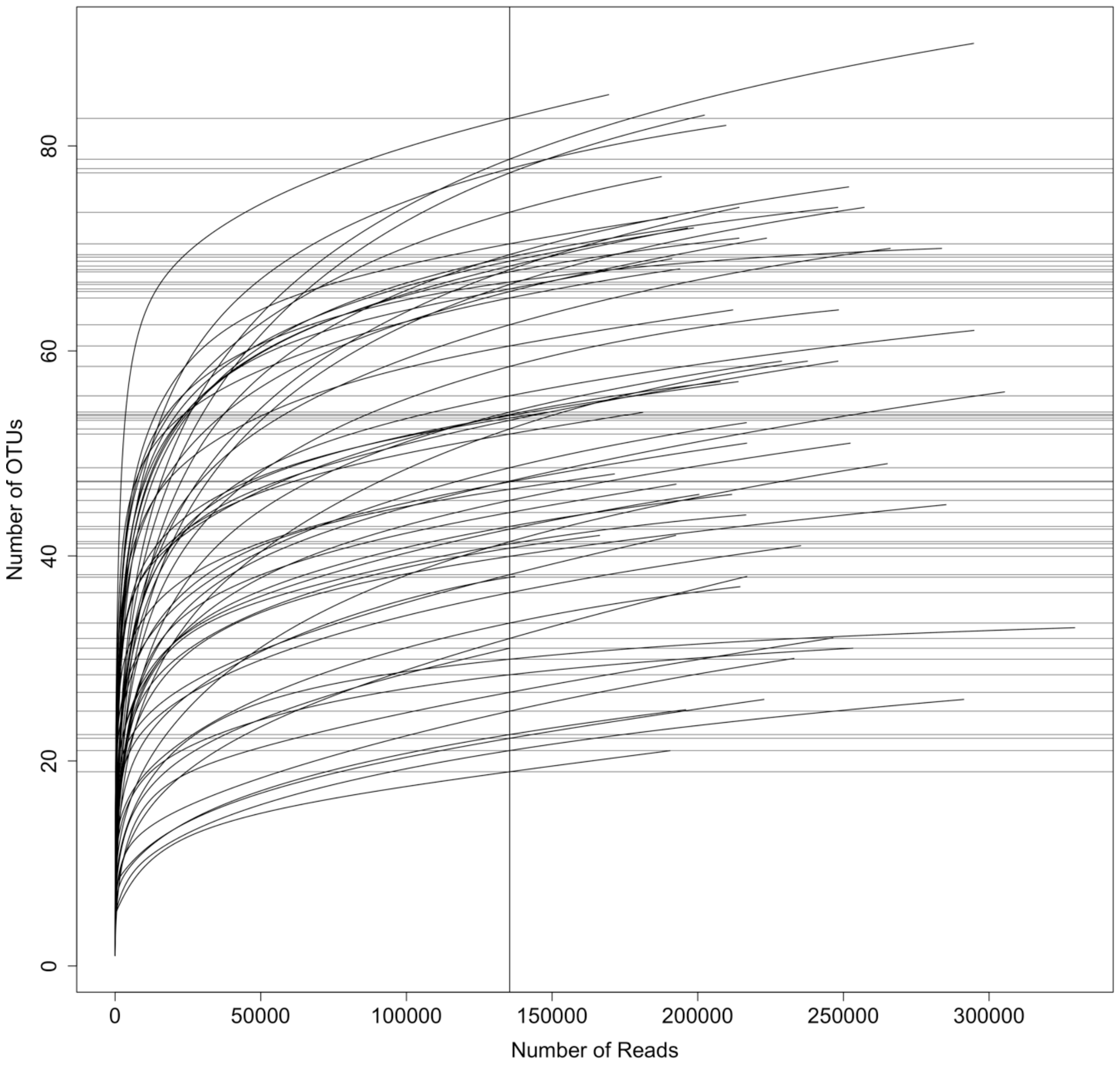

Supplementary Figure S1 Rarefaction curves of 54 samples representing number of OTUs against number of sequence reads. A vertical line is drawn at the smallest sequence count among samples with horizontal lines for the rarefied OTUs numbers.

Plant biodiversity assessment through pollen DNA metabarcoding in Natura 2000 habitats (Italian Alps) – Leontidou et al

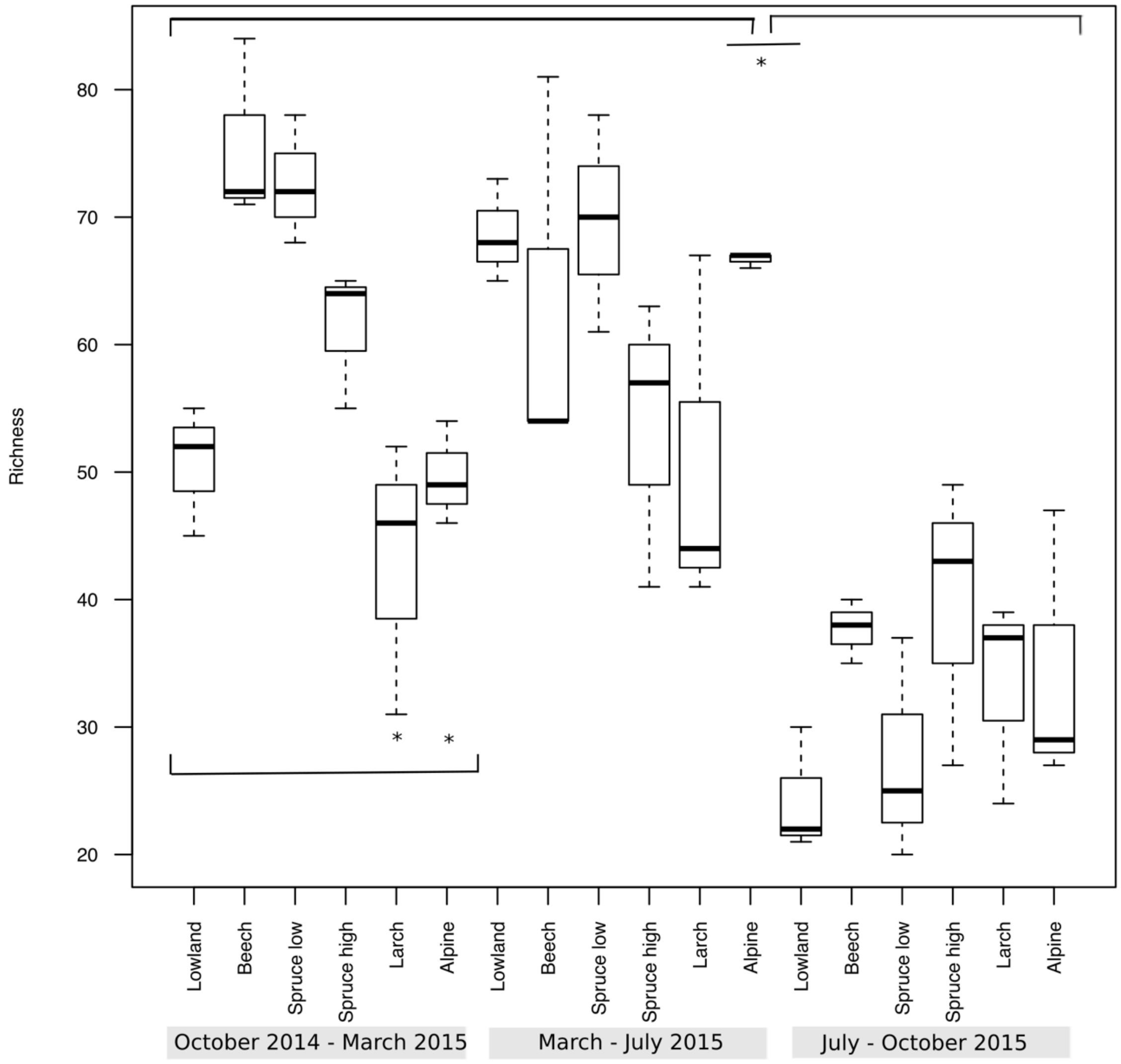

Supplementary Figure S2 Alpha diversity estimated as OTU richness of all samples grouped by habitat for each sampling period from low to high altitudes after DNA metabarcoding. The boxplots give the 25th and 75th percentile (bottom and top of the box, respectively) and the median (black thick line). The upper and lower whiskers correspond to the maximum and minimum values, respectively. The full names of the corresponding habitats are given in Table 1. Shown with asterisk are groups with statistically significant results.

Plant biodiversity assessment through pollen DNA metabarcoding in Natura 2000 habitats (Italian Alps) – Leontidou et al

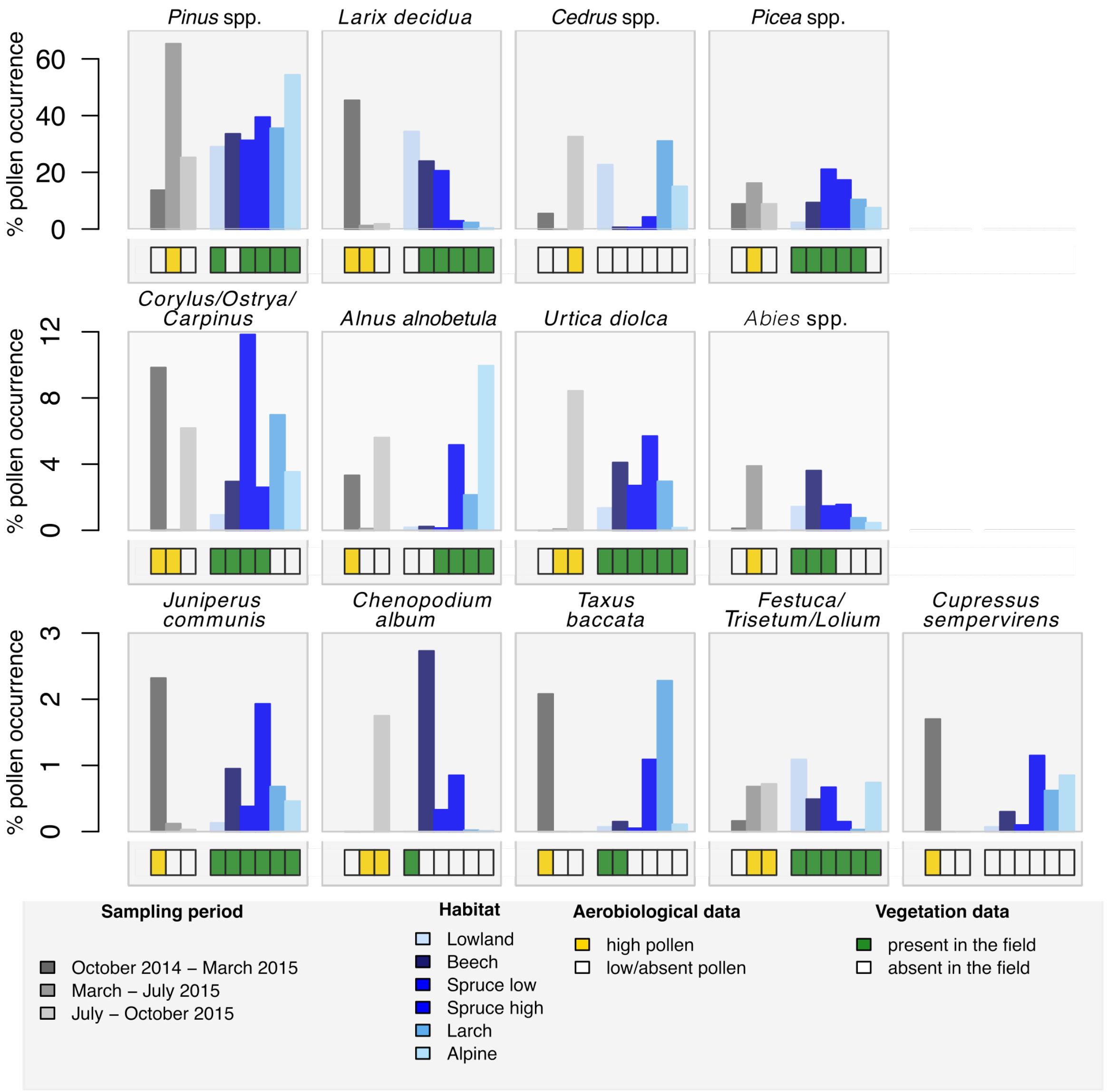

Supplementary Figure S3 Barplots representing relative read abundance of the plant taxa contributing ≥ 0.5% of the total number of reads per sampling period (upper left, grey part of each graph) and per habitat (upper right, coloured part of each graph) after HTS. For comparative purposes, the aerobiological and vegetation data shown at the bottom of each graph indicate when each taxon is represented in the airborne pollen with high pollen corresponding to concentration higher than 0.5 pollen grains per cubic meter of air (yellow squares) and where it occurs in the plant checklist (green squares). The full names of the habitat types are given in Table 1.

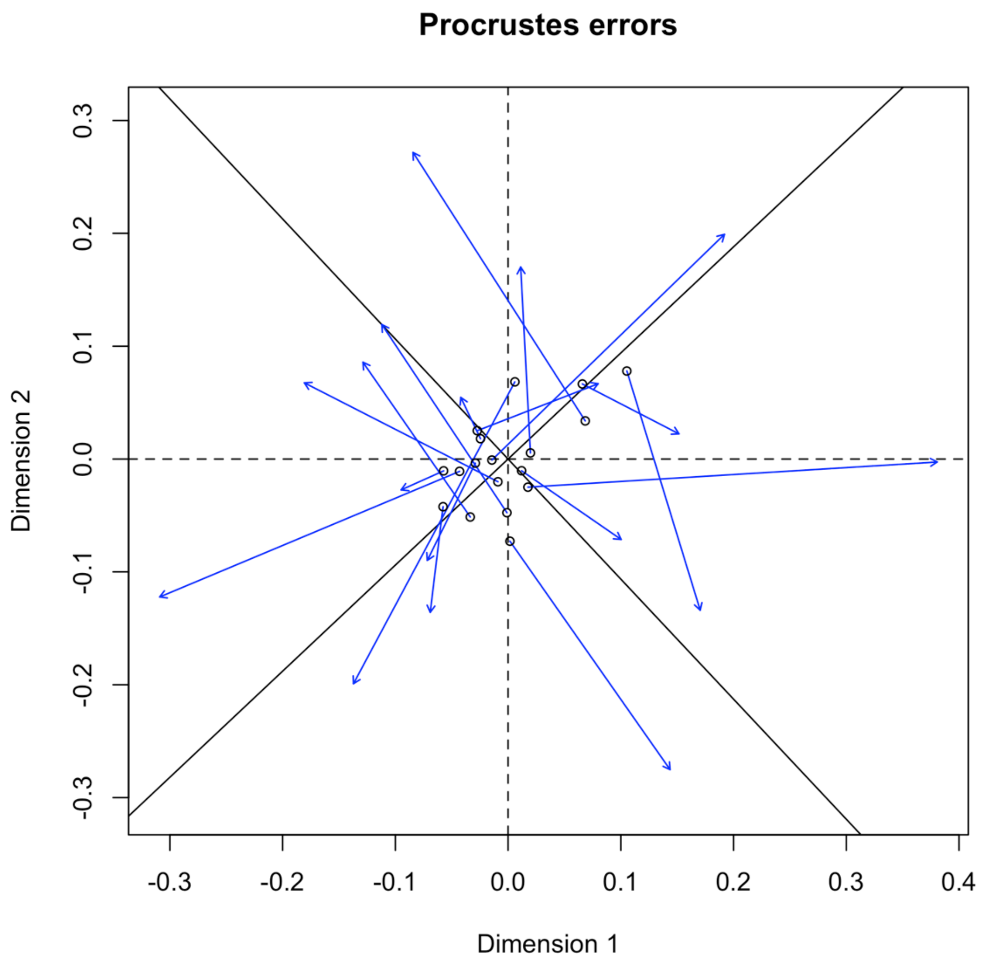

Supplementary Figure S4 Procrustes analysis comparing the NMDS ordination of HTS (circles) and microscope (arrow ends) samples, based on Jaccard dissimilarity values of beta diversity for the period March-July 2015.
